# An examination of public support for 35 nutrition interventions across seven countries

**DOI:** 10.1038/s41430-022-01211-5

**Published:** 2022-09-27

**Authors:** Simone Pettigrew, Leon Booth, Elizabeth Dunford, Tailane Scapin, Jacqui Webster, Jason Wu, Maoyi Tian, D. Praveen, Gary Sacks

**Affiliations:** 1 The George Institute for Global Health, Sydney, NSW Australia; 2 The University of New South Wales, Sydney, NSW Australia; 3 Department of Nutrition, Gillings Global School of Public Health, The University of North Carolina at Chapel Hill, Chapel Hill, NC USA; 4 The Global Centre for Preventive Health and Nutrition (GLOBE), Deakin University, Geelong, VIC Australia; 5 School of Public Health, Harbin Medical University, Harbin, China; 6 The George Institute for Global Health, New Delhi, India; 7 Prasanna School of Public Health, Manipal Academy of Higher Education, Manipal, India

**Keywords:** Obesity, Risk factors

## Abstract

**Background:**

Public support for evidence-based nutrition interventions can be an important determinant of government willingness to develop and implement such interventions. The aim of this study was to assess support for a broad range of nutrition interventions across seven countries: Australia, Canada, China, India, New Zealand, the United Kingdom, and the United States. Assessed interventions included those relating to food availability, affordability, reformulation, labelling, and promotion.

**Methods:**

Approximately 1000 adults per country (total *n* = 7559) completed an online survey assessing support for 35 nutrition interventions/policies. ANOVA analyses were used to identify differences between countries on overall levels of support and by intervention category. Multiple regression analyses assessed demographic and diet-related factors associated with higher levels of support across the total sample and by country.

**Results:**

Substantial levels of public support were found for the assessed interventions across the seven countries and five intervention categories. The highest levels were found in India (Mean across all interventions of 4.16 (standard deviation (SD) 0.65) on a 5-point scale) and the lowest in the United States (Mean = 3.48, SD = 0.83). Support was strongest for interventions involving food labelling (Mean = 4.20, SD = 0.79) and food reformulation (Mean = 4.17, SD = 0.87), and weakest for fiscal interventions (Mean = 3.52, SD = 1.06). Consumer characteristics associated with stronger support were higher self-rated health, higher educational attainment, female sex, older age, and perceptions of consuming a healthy diet.

**Conclusion:**

The results indicate substantial support for a large range of nutrition interventions across the assessed countries, and hence governments could potentially be more proactive in developing and implementing such initiatives.

## Introduction

A large and growing body of evidence provides support for a wide range of nutrition policies to assist in addressing burgeoning rates of obesity and other diet-related diseases globally [1–3]. Such policies include those relating to food availability, affordability, reformulation, labelling, and advertising [3–9]. Many of these policies involve enhancing the healthiness of the broader food environment rather than primarily focusing on encouraging individuals to change their behaviours within obesogenic environments that do not support healthy dietary choices [10]. This upstream approach is strongly endorsed by the world’s leading health agencies [9, 11].

The state of the evidence and the scale of diet-related health problems raises the question of why the policy approaches recommended by national and international health agencies are not being uniformly implemented by governments [12, 13]. Political appetite to implement effective food policy is understood to be a key determinant of whether recommended policies are introduced [14]. In turn, governments’ willingness to introduce such policies is reliant on numerous factors, one of which can be the extent to which the general public supports implementation [15]. As well as motivating governments via constituent sentiment, public support can (i) assist governments to resist industry opposition to policies that enhance public health but constrain market freedoms, (ii) enhance community compliance post-implementation, and (iii) inform decisions about the order in which specific interventions will be introduced and assist in the development of communications designed to address concerns among less supportive population segments [16–18].

To date, most research investigating public support for nutrition policies has focused on individual, high-income countries and examined a limited number of policies. Few studies have included low- and middle-income countries, and very little prior research has attempted to compare public support for the same policies cross-nationally [19–22]. Work to date suggests that levels of support can be dependent on policy characteristics, especially the level of perceived intrusiveness and consumer characteristics such as age and sex. Policies that involve greater restrictions have been found to be less popular than those that focus on information provision [20, 23], and females and older people are likely to be more supportive of nutrition policies than males and younger people [12, 15, 24]. Little is known about the extent to which these findings may be relevant to a broader range of potential nutrition policies and more diverse cultural contexts.

The aim of the present study was to extend current evidence relating to public support for nutrition policies by assessing support outcomes for a large range of interventions across a diverse range of countries. The included interventions represented five food policy categories that have been identified as being critical for achieving healthy diets at the population level [9, 25, 26]: availability, affordability, labelling, promotion, and reformulation. The included countries were Australia, Canada, China, India, New Zealand (NZ), the United Kingdom (UK), and the United States (US). Some of these countries have received very little research attention in the policy support literature to date (e.g., China, India, NZ), while others have been the subject of previous research and thus provide a comparison point for the results of this study [12, 20, 23, 24, 27].

## Methods

An ISO-accredited web panel provider (Pureprofile) was commissioned to recruit a minimum of 1,000 adults from each of the seven countries. This sample size was selected to enable within and between country analyses. As shown in Table 1, these countries exhibit variation across Hofstede’s cultural dimensions of individualism (the extent to which people prioritise themselves and their immediate families over the wider community), power distance (the extent to which there is unequal power distribution among members of society), and indulgence (the prioritisation of gratification over restraint) [28]. Each of these dimensions is likely to have implications for societal-level support for nutrition interventions that influence the food environment and affect people’s diets.

**Table 1:** Hofstede categorisation of selected cultural characteristics of included countries^a^.

	Australia	Canada	China	India	New Zealand	United Kingdom	UnitedStates
Individualism	90	80	20	48	79	89	91
Power distance	38	39	80	77	22	35	40
Indulgence	71	68	24	26	75	69	68

^a^Source: Hofstede Insights [28]. Individualism: the extent to which people prioritise themselves and their immediate families over the wider community; power distance: the extent to which there is unequal power distribution among members of society; indulgence: the prioritisation of gratification over restraint. All dimensions are measured on 100-point scales, with lower scores signifying lower individualism, power distance, and indulgence, respectively.

Quotas were applied to achieve samples within each country characterised by an approximately even number of males and females, an approximately even distribution across three age categories (18–34, 35–54, 55+ years), and at least two-thirds of the sample being in low and middle-income tertiles according to income distributions in each country. The latter requirement was designed to ensure appropriate representation of those on lower incomes who are often under-represented in survey research [29]. These quotas were met in most instances, notable exceptions being age distribution in India and income distribution in China. Likely reflecting the younger average age in India, only one in five respondents was in the 55+ years category. There were fewer low-income respondents from China compared to the other countries, compensated to some degree by a large proportion of middle-income participants. The sample profile (Table S1) and response rate data (Table S2) are provided in the supplementary materials. The study was approved by Curtin University Human Research Ethics Committee and respondents provided informed consent.

Respondents completed an online survey that included items on demographic characteristics, nutrition-related attitudes and behaviours, and extent of support for 35 nutrition interventions across the five topic areas of availability (*n* = 14 interventions), fiscal (*n* = 3), labelling (*n* = 5), promotion (*n* = 10), and reformulation (*n* = 3). The list of interventions was derived from recommendations commonly identified in key international papers and authoritative reports on policies for improving population diets (e.g., the NOURISHING database [11], the Lancet Commission on Obesity report [30], and the INFORMAS Food-EPI tool [31]). To optimise comparability across the proposed interventions, where possible the descriptions were kept neutral without specifying who would be responsible for implementation (e.g., government, food companies, other institutions) or how it would be implemented (e.g., through mandatory regulation, co-regulation, voluntary guidelines, local-level policy). Respondents indicated their support by selecting their level of agreement on a 5-point agreement scale (1 ‘Strongly disagree’ to 5 ‘Strongly agree’) for each intervention statement (see supplementary materials for item wording). Reflecting the differing policy environments in each country, the interventions were phrased in a manner for the agreement question to be relevant regardless of whether the intervention had already been implemented (e.g., ‘There should be regular public education campaigns about the importance of healthy eating’, ‘Supermarkets should be encouraged to promote healthy foods more heavily than unhealthy foods’). The survey instruments were presented in Mandarin and Hindi for respondents in China and India respectively, with English versions also made available for these respondents.

### Data analyses

Analyses were undertaken using SPSS 27 [32]. Descriptive analyses were performed to assess the level of support for each intervention and by intervention category within each country and overall. ANOVA analyses were used to determine whether the countries differed in terms of overall support for the interventions and each intervention category. Eight multivariate regression analyses were conducted to identify individual-level variables associated with support across the 35 assessed interventions for each country separately and all countries combined. The following independent variables were included in the regression analyses: age (continuous), sex (1 = male, 2 = female), household income (continuous), education (continuous), perceived diet healthiness (1 “I eat a very unhealthy diet” to 4 “I eat a very healthy diet”), self-rated health (1 “Poor” to 5 “Excellent”), and BMI (assessed from self-reported height and weight; continuous). Given the number of analyses conducted, a significance level of *p* < 0.001 was applied (two-tailed), and all test assumptions were met.

## Results

Overall, substantial levels of support were found across the seven countries and five intervention categories. The mean scores shown in Table 2 were all above the neutral scale midpoint of ‘3’, ranging from 3.04 (SD = 1.17) for fiscal interventions among US respondents to 4.27 (SD = 0.76) for labelling interventions among UK respondents. Of note was that the US respondents provided the lowest average scores for four of the five intervention categories and respondents from India the highest average scores for three. This was reflected in the US having the lowest ‘All initiatives’ composite mean (Mean = 3.48, SD = 0.83) and India the highest (Mean = 4.16, SD = 0.65).

**Table 2:** Support for diet-related policy intervention category by country^a^.

	*n*	Reformulation (*n* = 3 intervention types)		Labelling (*n* = 5 intervention types)		Fiscal (*n* = 3 intervention types)		Promotion (*n* = 10 intervention types)		Availability (*n* = 14 intervention types)		All initiatives^a^ (*n* = 35 intervention types)	
		M	SD	M	SD	M	SD	M	SD	M	SD	M	SD
Australia	1033	4.21^ab^	0.90	4.22^a^	0.85	3.33^a^	1.08	3.79^ab^	0.87	3.58^a^	0.89	3.76^a^	0.76
Canada	1079	4.26^b^	0.85	4.22^a^	0.79	3.31^a^	1.06	3.68^a^	0.89	3.56^a^	0.88	3.73^a^	0.76
China	1099	4.08^a^	0.67	4.17^a^	0.60	3.90^c^	0.70	4.05^c^	0.54	3.97^b^	0.54	4.03^c^	0.48
India	1086	4.12^ab^	0.92	4.17^a^	0.89	3.92^c^	0.94	4.20^d^	0.70	4.20^c^	0.71	4.16^d^	0.65
New Zealand	1090	4.17^ab^	0.88	4.18^a^	0.81	3.48^ab^	1.04	3.80^ab^	0.85	3.66^ad^	0.87	3.80^ab^	0.74
United Kingdom	1079	4.26^b^	0.87	4.27^a^	0.76	3.62^b^	1.03	3.93^bc^	0.82	3.74^d^	0.87	3.90^bc^	0.74
United States	1093	4.10^a^	0.94	4.14^a^	0.82	3.04^d^	1.17	3.38^e^	1.00	3.27^e^	0.99	3.48^e^	0.83
Total/average	7559	4.17	0.87	4.20	0.79	3.52	1.06	3.83	0.86	3.71	0.88	3.84	0.74

^a^Total of 35 nutrition interventions assessed on 5-point ‘1 = Strong disagree’ to ‘1 = Strongly agree’ scales. Countries with the same superscript letter did not differ significantly from each other at an alpha value of *p* < 0.001. M = mean, SD = standard deviation.

On average, the labelling intervention category had the highest mean score (Mean = 4.20, SD = 0.79), followed by the reformulation category (Mean = 4.17, SD = 0.87) (Table 2). The promotion (Mean = 3.83, SD = 0.86) and availability (Mean = 3.71, SD = 0.88) intervention categories were mid-range. The lowest scoring category was fiscal interventions, although the mean of 3.52 (SD = 1.06) was still above the neutral midpoint. As shown in Table 3, this lower overall support for fiscal interventions disguises a substantial difference between support in most countries for taxing unhealthy foods (Mean = 3.18, SD = 1.36) and beverages (Mean = 3.28, SD = 1.38) versus support for the subsidisation of fruit and vegetables (Mean = 4.09, SD = 1.12).

**Table 3:** Support for individual and aggregated reformulation, labelling, and fiscal interventions by country and overall.

Intervention	Australia (*n* = 1033)		Canada (*n* = 1079)		China(n = 1099)		India (*n* = 1086)		New Zealand (*n* = 1090)		United Kingdom (*n* = 1079)		United States (*n* = 1093)		Overall (*n* = 7559)	
	%^a^	M (SD)	%^a^	M (SD)	%^a^	M (SD)	%^a^	M (SD)	%^a^	M (SD)	%^a^	M(SD)	%^a^	M (SD)	%^a^	M (SD)
***Reformulation***																
Manufacturers should reduce the amount of added sugar in their products	78	4.25 (1.01)	79	4.26 (0.98)	79	4.08 (0.83)	77	4.16 (1.08)	81	4.29 (0.97)	82	4.29 (0.96)	74	4.13 (1.05)	79	4.21 (0.99)
Manufacturers should reduce the amount of salt in their products	75	4.15 (0.99)	79	4.24 (0.96)	77	4.07 (0.83)	74	4.04 (1.06)	73	4.09 (0.96)	78	4.23 (0.98)	71	4.05 (1.07)	75	4.12 (0.98)
Manufacturers should reduce the amount of saturated fat in their products	77	4.22 (0.96)	80	4.27 (0.91)	78	4.08 (0.83)	78	4.16 (1.06)	75	4.13 (0.96)	81	4.27 (0.92)	75	4.11 (1.04)	78	4.18 (0.96)
*Reformulation composite*	*77*	*4.21 (0.90)*	*79*	*4.26 (0.85)*	*78*	*4.08 (0.67)*	*76*	*4.12 (0.92)*	*76*	*4.17 (0.88)*	*80*	*4.26 (0.87)*	*73*	*4.10 (0.94)*	*77*	*4.17 (0.87)*
***Labelling***																
The amount of added sugar in a packaged food should be reported on the label	80	4.31 (1.05)	83	4.36 (0.99)	84	4.17 (0.86)	76	4.14 (1.23)	82	4.34 (1.02)	82	4.33 (0.96)	81	4.26 (1.05)	81	4.27 (1.03)
The amount of trans fat in a packaged food should be reported on the label	77	4.24 (1.01)	83	4.37 (0.90)	79	4.14 (0.86)	77	4.12 (1.13)	78	4.20 (0.99)	79	4.27 (0.94)	80	4.26 (0.97)	79	4.23 (0.98)
The calories/kilojoules content of food and drink should be displayed on menus at fast food outlets	77	4.21 (1.01)	77	4.19 (1.01)	76	4.06 (0.85)	78	4.17 (1.11)	69	4.02 (1.04)	79	4.25 (0.94)	79	4.22 (0.99)	77	4.16 (1.00)
Foods that are especially high in sugar, fat, or salt should have health warnings on them	76	4.18 (1.05)	74	4.13 (1.05)	84	4.25 (0.83)	80	4.22 (1.11)	76	4.16 (1.02)	79	4.22 (0.95)	67	3.95 (1.15)	77	4.16 (1.03)
There should be a simple indicator of the product’s healthiness shown on the front of the pack	78	4.19 (0.99)	73	4.07 (0.99)	84	4.22 (0.78)	80	4.21 (1.02)	78	4.17 (0.97)	82	4.26 (0.90)	72	4.02 (1.06)	78	4.16 (0.97)
*Labelling composite*	*78*	*4.22 (0.85)*	*78*	*4.22 (0.79)*	*81*	*4.17 (0.60)*	*78*	*4.17 (0.89)*	*77*	*4.18 (0.81)*	*80*	*4.27 (0.76)*	*76*	*4.14 (0.82)*	*78*	*4.20 (0.79)*
***Fiscal***																
There should be a tax on sugary drinks to reduce consumption	40	3.05 (1.43)	37	3.00 (1.41)	65	3.72 (0.94)	63	3.75 (1.26)	47	3.26 (1.44)	53	3.44 (1.34)	32	2.71 (1.47)	48	3.28 (1.38)
There should be a tax on fat in food products to reduce consumption	34	2.91 (1.39)	33	2.86 (1.37)	66	3.82 (0.94)	64	3.77 (1.2)	35	2.93 (1.33)	49	3.35 (1.31)	29	2.62 (1.43)	44	3.18 (1.36)
Fruit and vegetables should be subsidised to make them more affordable	72	4.02 (1.14)	73	4.09 (1.12)	79	4.17 (0.86)	80	4.25 (1.04)	79	4.23 (1.14)	74	4.07 (1.12)	64	3.79 (1.32)	74	4.09 (1.12)
*Fiscal composite*	*49*	*3.33 (1.08)*	*48*	*3.31 (1.06)*	*70*	*3.90 (0.70)*	*69*	*3.92 (0.94)*	*54*	*3.48 (1.04)*	*59*	*3.62 (1.03)*	*42*	*3.04 (1.17)*	*55*	*3.52 (1.06)*

^a^Proportion of respondents selecting response options 4 or 5 on a 5-point ‘1 = Strong disagree’ to ‘5 = Strongly agree’ scale. M = mean, SD = standard deviation.

Italic values denote composite data.

At the individual intervention level, 13 of the 35 interventions were actively supported (i.e., by selecting ‘Agree’ or ‘Strongly agree’ on the 5-point agreement scale) by 75% or more respondents across the total sample (Tables 3–5). Three interventions received 75 + % support in the seven countries individually as well as overall, each of which related to specific nutrients: ‘The amount of added sugar in a packaged food should be reported on the label’, ‘The amount of trans fat in a packaged food should be reported on the label’, and ‘Manufacturers should reduce the amount of saturated fat in their products’. The highest levels of support for specific interventions within individual countries were found for ‘There should be regular public education campaigns about the importance of healthy eating’ (87% India, 86% China) and ‘Hospitals should provide only healthy foods to patients’ (87% India).

**Table 4:** Support for individual and aggregated promotion interventions by country and overall.

Intervention	Australia (*n* = 1033)		Canada (*n* = 1079)		China (*n* = 1099)		India (*n* = 1086)		New Zealand (*n* = 1090)		United Kingdom (*n* = 1079)		United States (*n* = 1093)		Overall (*n* = 7559)	
	%^a^	M (SD)	%^a^	M (SD)	%^a^	M (SD)	%^a^	M (SD)	%^a^	M (SD)	%^a^	M (SD)	%^a^	M (SD)	%^a^	M (SD)
Supermarkets should be encouraged to promote healthy foods more heavily than unhealthy foods	74	4.12 (1.01)	72	4.08 (1.03)	82	4.20 (0.81)	81	4.3 (1.02)	75	4.14 (1.03)	77	4.16 (0.99)	63	3.82 (1.23)	75	4.12 (1.03)
Healthy foods should be featured on end-of-aisle displays in supermarkets rather than unhealthy foods	71	4.05 (1.02)	69	4.00 (1.04)	81	4.13 (0.81)	81	4.23 (0.99)	71	4.06 (1.02)	75	4.11 (1.02)	60	3.76 (1.23)	73	4.05 (1.03)
Unhealthy foods should be removed from supermarket check-out areas	55	3.63 (1.25)	44	3.33 (1.30)	71	3.91 (0.93)	69	3.97 (1.16)	50	3.53 (1.28)	60	3.72 (1.26)	37	3.00 (1.42)	55	3.58 (1.28)
Meal deals for children should have healthy options as the automatic default options	73	4.07 (1.00)	76	4.15 (0.91)	83	4.14 (0.80)	80	4.2 (1.06)	76	4.13 (0.96)	80	4.22 (0.91)	70	3.98 (1.10)	77	4.13 (0.97)
Junk food billboard advertising should not be allowed near schools or other children’s settings	56	3.68 (1.19)	48	3.41 (1.27)	75	4.01 (0.91)	74	4.07 (1.1)	56	3.64 (1.21)	67	3.91 (1.13)	39	3.01 (1.40)	59	3.68 (1.23)
There should be regular public education campaigns about the importance of healthy eating	71	3.98 (1.03)	72	4.05 (0.95)	86	4.20 (0.71)	87	4.41 (0.87)	72	4.03 (0.99)	72	4.01 (0.94)	66	3.88 (1.07)	75	4.08 (0.96)
Junk food advertising should not be allowed on government-owned properties	55	3.61 (1.22)	46	3.41 (1.26)	72	3.93 (0.88)	75	4.13 (1.04)	55	3.65 (1.21)	57	3.71 (1.14)	39	3.11 (1.38)	57	3.65 (1.21)
Unhealthy food sponsorships should be removed from elite/professional sport	54	3.56 (1.26)	46	3.35 (1.28)	72	3.95 (0.90)	77	4.19 (1.01)	54	3.60 (1.24)	61	3.76 (1.17)	38	3.03 (1.39)	57	3.63 (1.24)
Unhealthy food sponsorships should be removed from community sporting clubs (e.g. local football or basketball clubs)	52	3.50 (1.25)	46	3.37 (1.27)	72	3.91 (0.89)	76	4.16 (1.02)	49	3.50 (1.22)	58	3.70 (1.16)	37	3.02 (1.39)	56	3.6 (1.23)
Unhealthy food sponsorships should be removed from children’s sporting activities	59	3.72 (1.22)	56	3.64 (1.25)	77	4.11 (0.86)	82	4.33 (0.94)	60	3.78 (1.21)	68	3.95 (1.11)	45	3.25 (1.38)	64	3.83 (1.2)
*Promotion composite*	*62*	*3.79 (0.87)*	*58*	*3.68 (0.89)*	*77*	*4.05 (0.54)*	*78*	*4.20 (0.70)*	*62*	*3.80 (0.85)*	*68*	*3.93 (0.82)*	*49*	*3.38 (1.00)*	*65*	*3.83 (0.86)*

Italic values denote composite data.

^a^Proportion of respondents selecting response options 4 or 5 on a 5-point ‘1 = Strong disagree’ to ‘5 = Strongly agree’ scale. M = mean, SD = standard deviation.

**Table 5:** Support for individual and aggregated availability interventions by country and overall.

Intervention	Australia (*n* = 1033)		Canada (*n* = 1079)		China (*n* = 1099)		India (*n* = 1086)		New Zealand (*n* = 1090)		United Kingdom (*n* = 1079)		United States (*n* = 1093)		Overall (*n* = 7559)	
	%^a^	M (SD)	%^a^	M (SD)	%^a^	M (SD)	%^a^	M (SD)	%^a^	M (SD)	%^a^	M (SD)	%^a^	M (SD)	%^a^	M (SD)
Fast food chains should not be able to open new outlets near schools or other children’s settings	51	3.55 (1.23)	44	3.29 (1.30)	67	3.87 (0.93)	72	4.02 (1.1)	50	3.50 (1.25)	62	3.79 (1.18)	35	2.91 (1.39)	54	3.56 (1.25)
Junk food should not be sold in places where children are doing sport	55	3.64 (1.22)	51	3.49 (1.28)	78	4.13 (0.89)	75	4.12 (1.08)	53	3.60 (1.23)	64	3.85 (1.16)	42	3.11 (1.42)	60	3.71 (1.24)
School canteens should be allowed to sell only healthy foods	53	3.57 (1.20)	58	3.69 (1.15)	78	4.11 (0.89)	76	4.26 (1.01)	56	3.64 (1.16)	60	3.72 (1.16)	49	3.37 (1.34)	62	3.77 (1.17)
Vending machines containing unhealthy foods should not be allowed in schools	62	3.81 (1.18)	57	3.66 (1.22)	75	4.02 (0.90)	78	4.2 (1.03)	65	3.86 (1.16)	67	3.90 (1.13)	49	3.33 (1.40)	65	3.83 (1.18)
Child care centres should provide only healthy food options	73	4.07 (1.04)	72	4.00 (1.05)	81	4.19 (0.86)	83	4.31 (0.98)	72	4.05 (1.04)	70	3.97 (1.04)	64	3.75 (1.23)	73	4.05 (1.05)
Hospitals should provide only healthy foods to patients	73	4.03 (1.04)	79	4.18 (0.97)	80	4.08 (0.83)	87	4.5 (0.94)	78	4.19 (0.97)	76	4.11 (0.97)	72	4.01 (1.06)	78	4.16 (0.98)
Vending machines containing unhealthy foods should not be allowed in hospitals	48	3.43 (1.24)	47	3.47 (1.21)	70	3.96 (0.96)	80	4.26 (1.03)	52	3.63 (1.17)	58	3.70 (1.19)	40	3.15 (1.32)	57	3.66 (1.21)
Vending machines containing sugary drinks should not be allowed in hospitals	48	3.41 (1.25)	46	3.42 (1.23)	57	3.59 (0.98)	74	4.07 (1.04)	54	3.65 (1.20)	56	3.64 (1.20)	37	3.06 (1.33)	53	3.55 (1.22)
Universities should ensure healthy food options are available to students	73	4.04 (1.03)	77	4.18 (0.97)	83	4.18 (0.80)	82	4.3 (0.96)	78	4.18 (0.95)	78	4.13 (0.94)	74	4.10 (1.06)	78	4.16 (0.96)
Vending machines containing unhealthy foods should not be allowed in universities	36	3.09 (1.22)	35	3.09 (1.25)	61	3.69 (1.02)	73	4.07 (1.08)	37	3.19 (1.21)	43	3.32 (1.22)	30	2.82 (1.35)	45	3.33 (1.26)
Employers should ensure that workplace food options are primarily healthy	55	3.59 (1.14)	59	3.70 (1.11)	78	4.06 (0.81)	80	4.2 (1.01)	58	3.67 (1.12)	65	3.82 (1.02)	50	3.46 (1.20)	64	3.79 (1.09)
Vending machines containing unhealthy foods should not be allowed in workplaces	33	3.01 (1.23)	32	2.97 (1.26)	64	3.75 (0.98)	73	4.05 (1.05)	32	3.08 (1.22)	40	3.26 (1.21)	28	2.75 (1.35)	43	3.27 (1.27)
Sporting venues should ensure that foods available for purchase are primarily healthy	56	3.64 (1.13)	55	3.59 (1.16)	79	4.12 (0.84)	81	4.25 (1)	61	3.75 (1.07)	64	3.83 (1.07)	41	3.23 (1.29)	62	3.77 (1.13)
Vending machines containing unhealthy foods should not be allowed in sporting venues	39	3.17 (1.25)	36	3.06 (1.27)	69	3.85 (0.91)	77	4.16 (1.03)	40	3.27 (1.19)	46	3.40 (1.22)	28	2.72 (1.37)	48	3.38 (1.27)
*Availability composite*	*54*	*3.58 (0.89)*	*53*	*3.56 (0.88)*	*73*	*3.97 (0.54)*	*78*	*4.20 (0.71)*	*56*	*3.66 (0.87)*	*61*	*3.74 (0.87)*	*46*	*3.27 (0.99)*	*60*	*3.71 (0.88)*

Italic values denote composite data.

^a^Proportion of respondents selecting response options 4 or 5 on a 5-point ‘1 = Strong disagree’ to ‘5 = Strongly agree’ scale. M = mean, SD = standard deviation.

For most countries, there were few instances of only a minority of respondents expressing support for individual interventions. The notable exception was the US, where <50% of respondents supported 18 of the 35 assessed interventions. Individual interventions receiving the lowest levels of support were those relating to taxing unhealthy foods and beverages and restricting the products available in vending machines in locations such as workplaces, universities, and sporting venues (vending machines in hospitals and schools received majority support overall, albeit not among some individual countries).

The vending machine interventions were also the most polarising across countries. For example, ‘Vending machines containing unhealthy foods should not be allowed in sporting venues’ received support from 28% of respondents in the US versus 77% in India. The interventions with the most similar responses across countries were those relating to the reporting of saturated fat (Australia and India 77% vs Canada 83%) and trans fat (NZ and the US 75% vs 81% UK) on product labels.

The regression analyses results are presented in Table 6. Across the total sample, while most of the assessed variables were significantly associated with overall support, the largest effect sizes were found for higher self-rated health (β = 0.17) and educational attainment (β = 0.12). There were some notable country-specific differences. For example, female sex was a significant predictor of overall support in only Australia (β = 0.16), Canada (β = 0.11), and NZ (β = 0.14), and education was only significant for Australia (β = 0.11), China (β = 0.20), and India (β = 0.12). Perceived diet healthiness was related to policy support in all countries except China and India. BMI was not associated with level of support in any of the individual countries.

**Table 6:** Regression analyses examining factors associated with overall support score by country and total sample.

	Australia (*n* = 1026)	Canada (*n* = 1073)	China (*n* = 1093)	India (*n* = 1076)	New Zealand (*n* = 1068)	United Kingdom (*n* = 1062)	United States (*n* = 1090)	All (*n* = 7488)
	β [SE]	β [SE]	β [SE]	β [SE]	β [SE]	β [SE]	β [SE]	β [SE]
Age (older)	0.15 [<0.01]*	0.12 [<0.01]*	0.02 [<0.01]	0.13 [<0.01]*	0.11 [<0.01]*	0.16 [<0.01]*	−0.04 [<0.01]	0.08 [<0.01]*
Sex (Female)	0.16 [0.05]*	0.11 [0.05]*	−0.01 [0.03]	0.06 [0.04]	0.14 [0.05]*	0.07 [0.04]	0.06 [0.05]	0.08 [0.02]*
Household Income	0.02 [0.03]	−0.01 [0.03]	−0.12 [0.02]*	0.06 [0.03]	−0.04 [0.03]	−0.02 [0.03]	<0.01 [0.04]	<0.01 [0.01]
Education	0.11 [0.01]*	0.04 [0.01]	0.20 [0.01]*	0.12 [0.01]*	0.09 [0.01]	0.06 [0.01]	<0.01 [0.01]	0.12 [0.01]*
Higher perceived healthiness of diet	0.15 [0.04]*	0.20 [0.04]*	0.08 [0.03]	−0.04 [0.03]	0.15 [0.05]*	0.34 [0.04]*	0.26 [0.04]*	0.04 [0.01]*
Higher self−rated health	0.05 [0.03]	0.11 [0.03]	0.13 [0.02]*	0.22 [0.02]*	0.01 [0.03]	0.05 [0.02]	0.10 [0.03]	0.17 [0.01]*
BMI	−0.01 [<0.01]	0.05 [<0.01]	−0.01 [<0.01]	0.04 [<0.01]	−0.04 [<0.01]	0.02 [<0.01]	<0.01 [<0.01]	−0.03 [<0.01]

*p < 0.001.

Eight regression models were conducted to assess relationships at country level and for the total data set. Respondents who answered ‘prefer not to say’ for education were excluded, resulting in slightly lower n values for this variable. Standardised beta coefficients (β) are reported with values standardised according to the units of measurement. SE = standard error, BMI = body mass index.

## Discussion

This study across a diverse range of countries found majority support for most of the nutrition interventions examined. Consistent with the cultural dimensions outlined in Table 1, support was strongest in India and China and weakest in the US. India and China score high on power distance (indicating acceptance of authority), low on indulgence (suggesting a willingness to forego personal gratification), and low individualism (prioritising the well-being of the broader community). By comparison, the US is at the opposite end of the spectrum on these dimensions, which is reflected in the results of this and previous research examining public support for health-related interventions [17, 20, 22]. Cultural dimensions therefore appear to be a useful lens through which to assess uptake of nutrition policies [19]. However, it is noteworthy that Australia and the UK, for example, are high on individualism and indulgence, and NZ is especially low on power distance, yet respondents in these countries demonstrated markedly higher support for many of the interventions compared to the US. These findings may reflect the current food policy environments within these countries and the extent to which the populations are already accustomed (or not) to more interventionist nutrition policy (for country-specific information on current food policy contexts see the NOURISHING Framework [11]).

Similar to previous research, the interventions receiving the highest levels of support were those relating to labelling and reformulation [19, 21, 23, 33]. These approaches to improving the quality of the food supply involve the provision of information and modification of product content by manufacturers, and therefore are relatively low in terms of intrusiveness on consumers. By comparison, the interventions with the lowest levels of support were those pertaining to taxes on unhealthy foods and beverages (fiscal) and the contents of vending machines (availability), both of which have more direct impacts on consumers. The results relating to promotion interventions were generally mid-range, with those requiring the promotion of healthier options rated higher than those restricting the promotion of unhealthy options. These outcomes are consistent with previous work finding level of policy intrusiveness to be inversely related to level of support [19, 20, 34], highlighting the need to increase the public’s understanding of the importance and effectiveness of more intrusive interventions.

The lack of popularity of taxes has been noted elsewhere [12, 21, 24, 33], and policy makers face clear challenges in presenting this form of intervention to the general public in a manner that fosters greater acceptance. Taxes are also strongly opposed by the food industry [35], making it even more important to gain public approval to assist in overcoming the influence of industry lobbyists. Previous research has identified explicit hypothecation of food tax revenue to address health-related issues as a possible means of overcoming resistance among substantial sections of the population [24, 27]. This was not tested in the present study and constitutes an important focus for future research given the substantial and growing evidence that fiscal interventions have a key role to play in effective food policy [3–5, 9].

The finding of lower support for restrictions on vending machines is also worthy of further investigation. There is a noted lack of evidence relating to the way in which consumers across different countries respond to the idea of healthy vending machines [33]. Many people may rely on these food sources in locations (e.g., workplaces and education institutions) and at times (e.g., after hours) when other options are not readily available [36, 37]. In such situations, restricting the contents of vending machines may be construed as effectively eradicating choice, and more work may be needed across countries to increase consumer receptivity to such interventions.

Consistent with previous research, demographic factors such as being female, older, and more educated were significantly associated with higher intervention support levels across the total sample [12, 15, 24]. However, as per the limited prior work comparing predictors of support internationally [20, 22], these associations differed somewhat between countries. For example, older respondents were found to be more supportive of the assessed interventions in all countries except China and the US; females were more likely to express support in Australia, Canada, and NZ, but not the other four countries; and education level was only significant as a predictor of support in Australia, China, and India. It is therefore important to understand the role of demographic predictors in individual countries when assessing policy support.

The high levels of support found for most interventions in most countries indicates governments have high levels of support from their populations to take a more proactive stance in nutrition policy development and implementation. This in turn raises the issue of why evidence-based interventions with strong community support are not already in place. A well-recognised contributor to this situation is vigorous resistance from the food industry [13, 35, 38, 39], highlighting the importance of countries applying appropriate strategies and protocols to minimise industry interference in policy making. A further consideration is the logistical complexity associated with introducing and monitoring policies that require actions across sectors, such as those involving taxation, institutional food provisioning, and communications regulation [40]. Even just within the public sector, working towards integrated food policy will require coordination between government departments, including those that are unaccustomed to prioritising health, hence requiring strong political commitment [14].

The primary strength of this study was the involvement of sizeable samples recruited from seven countries, including those with varying cultural profiles and different levels of economic development. Such cross-cultural analyses enable observation of similarities and differences that can provide insights into potential leverage points for ongoing improvements in countries’ nutrition policies. The results can constitute groundwork for examining changes over time in response to differing patterns of intervention implementation.

An important limitation was use of a web panel provider for data collection and the resulting non-probability sample that is likely to have differed on some characteristics compared to the populations from which it was drawn. This is especially notable for China and India, where web panel use would have excluded involvement of those with lower literacy levels. Future research could use alternative participant recruitment methods to access a broader range of respondents. In addition, due to the number of interventions and countries included in the study, no attempt was made to account for the policy environments in each country and the associated differing levels of familiarity with the assessed interventions. This is an important area of future research as it would enable identification of how support changes at different stages of policy implementation. Another useful approach would be experiments to explore effective methods of communicating with the public about the benefits of nutrition policies to increase support, especially among less receptive population subgroups [15]. Finally, future work comparing policy support across countries would ideally include a larger number of low- and middle-income countries to provide greater insights into these important food policy contexts.

In conclusion, the results of this study indicate that populations across various countries are supportive of a large range of nutrition policies, and hence that governments can potentially be more proactive in this space. However, focused efforts are needed to increase support for some evidence-based policies, especially those relating to the application of taxes to unhealthy food products.

## Supplementary information

Supplementary materials

## Data Availability

The data are available from the first author upon reasonable request for non-commercial use.
