# Supplementary material for: An examination of public support for 35 nutrition interventions across seven countries
Source: Eur J Clin Nutr. 2022 Sep 27;77(2):235–45. doi: 10.1038/s41430-022-01211-5 (PMC9908538; doi:10.1038/s41430-022-01211-5)
Supplement: Supplementary file 1 — Supplementary materials [file 41430_2022_1211_MOESM1_ESM.docx]

**Public support for 35 nutrition interventions across seven countries**

**Supplementary materials**

*Supplementary Table S1: Sample profile*

|  | **Australia**  **(n=1033)** | **Canada**  **(n=1079)** | **China**  **(n=1099)** | **India**  **(n=1086)** | **New Zealand**  **(n=1090)** | **United Kingdom**  **(n=1079)** | **United States**  **(n=1093)** | **Total sample**  **(n=7559)** |
| --- | --- | --- | --- | --- | --- | --- | --- | --- |
|  | % | % | % | % | % | % | % | % |
| Respondent characteristics | |  |  |  |  |  |  |  |
| Sex |  |  |  |  |  |  |  |  |
| Female | 51 | 51 | 49 | 48 | 52 | 51 | 51 | 50 |
| Male | 49 | 49 | 51 | 52 | 48 | 49 | 49 | 50 |
| Age (years) |  |  |  |  |  |  |  |  |
| 18-34 | 31 | 29 | 32 | 48 | 30 | 29 | 30 | 33 |
| 35-54 | 34 | 34 | 42 | 34 | 39 | 35 | 34 | 36 |
| 55+ | 35 | 37 | 26 | 18 | 31 | 36 | 36 | 31 |
| Household income |  |  |  |  |  |  |  |  |
| Low | 34 | 33 | 13 | 29 | 28 | 33 | 42 | 29 |
| Mid | 36 | 49 | 63 | 58 | 42 | 28 | 41 | 46 |
| High | 30 | 18 | 24 | 13 | 30 | 40 | 16 | 25 |

Note: Percentages may not add to 100% due to rounding

**Response rate data**

Supplementary Table S2 provides the response rates by country. Screened out respondents were those who failed to meet the eligibility criterion of being 18 years of age or older, and disqualified respondents were those who engaged in behaviors that could affect the quality of the data (e.g., completed the survey at a rapid speed that is not conducive to properly reading the items or completed multiple surveys from the same device). Those in the incomplete category failed to complete the entire survey.

*Supplementary Table S2: Response rates by country*

|  | **Australia** | **Canada** |  | **China** | **India** | **NZ** | **UK** | **US** |
| --- | --- | --- | --- | --- | --- | --- | --- | --- |
| Starts^ | 1411 | 1328 |  | 1864 | 1970 | 1226 | 1263 | 1540 |
| Completes | 1035 | 1079 |  | 1099 | 1131 | 1090 | 1079 | 1093 |
| Screeners | 8 | 26 |  | 31 | 70 | 2 | 11 | 19 |
| Quota Full | 305 | 100 |  | 425 | 717 | 95 | 37 | 257 |
| Incompletes | 63 | 123 |  | 309 | 52 | 39 | 136 | 171 |

^This category includes individuals who elected to commence the survey that was promoted to them within the Pureprofile app via a generic ad that did not specify the survey topic.

**Survey item wording (English version)**

**Demographic variables**

- How old are you? Please write your age in years in the space below.
- Are you?

Male

Female

Other

Prefer not to say

- What is your annual household income?

Less than $50,000 (amounts differed by country)

$50,000 - $100,000

More than $100,000

- What is the highest level of education you have completed? (*wording of response options varied by country according to local education systems but number of response options was consistent*)

Year 11 or below

Year 12

Vocational qualification (e.g. trade/apprenticeship)

Other TAFE or technical certificate

Diploma

Bachelor degree (including Honours)

Post graduate diploma or degree

Other (please specify)

Prefer not to say

- What is your weight?

KG ______ OR Stones _____ Pounds _____

- What is your height?

Metres _____ OR Feet _____ Inches _____

**Attitudinal variables**

How would you describe your diet?

- I eat a very healthy diet
- I eat a mostly healthy diet
- I eat a mostly unhealthy diet
- I eat a very unhealthy diet

In general, would you say that your health is poor, fair, good, very good, or excellent?

- Poor
- Fair
- Good
- Very good
- Excellent

**Intervention support variables**

To what extent do you agree or disagree with each of the following?

1 2 3 4 5

Strongly disagree Strongly agree

- The amount of added sugar in a packaged food should be reported on the label
- The amount of trans fat in a packaged food should be reported on the label
- The calories/kilojoules content of food and drink should be displayed on menus at fast food outlets
- Foods that are especially high in sugar, fat, or salt should have health warnings on them
- There should be a simple indicator of the product’s healthiness shown on the front of the pack
- There should be a tax on sugary drinks to reduce consumption
- There should be a tax on fat in food products to reduce consumption
- Fruit and vegetables should be subsidised to make them more affordable
- Supermarkets should be encouraged to promote healthy foods more heavily than unhealthy foods
- Healthy foods should be featured on end-of-aisle displays in supermarkets rather than unhealthy foods
- Unhealthy foods should be removed from supermarket check-out areas
- Meal deals for children should have healthy options as the automatic default options
- Fast food chains should not be able to open new outlets near schools or other children’s settings
- Junk food billboard advertising should not be allowed near schools or other children’s settings
- Junk food should not be sold in places where children are doing sport
- School canteens should be allowed to sell only healthy foods
- Vending machines containing unhealthy foods should not be allowed in schools
- Child care centres should provide only healthy food options
- Hospitals should provide only healthy foods to patients
- Vending machines containing unhealthy foods should not be allowed in hospitals
- Vending machines containing sugary drinks should not be allowed in hospitals
- Universities should ensure healthy food options are available to students
- Vending machines containing unhealthy foods should not be allowed in universities
- Employers should ensure that workplace food options are primarily healthy
- Vending machines containing unhealthy foods should not be allowed in workplaces
- Sporting venues should ensure that foods available for purchase are primarily healthy
- Vending machines containing unhealthy foods should not be allowed in sporting venues
- There should be regular public education campaigns about the importance of healthy eating
- Junk food advertising should not be allowed on government-owned properties (e.g., buses, bus stops, and government buildings)
- Unhealthy food sponsorships should be removed from elite/professional sport
- Unhealthy food sponsorships should be removed from community sporting clubs (e.g., local football or basketball clubs)
- Unhealthy food sponsorships should be removed from children’s sporting activities
- Manufacturers should reduce the amount of added sugar in their products
- Manufacturers should reduce the amount of salt in their products
- Manufacturers should reduce the amount of saturated fat in their products
